# Presaccadic Attention Shifts Up‐ and Downwards: Evidence From the Pupil Light Response

**DOI:** 10.1111/psyp.70047

**Published:** 2025-03-17

**Authors:** Damian Koevoet, Marnix Naber, Christoph Strauch, Stefan Van der Stigchel

**Affiliations:** ^1^ Experimental Psychology, Helmholtz Institute Utrecht University Utrecht the Netherlands

**Keywords:** presaccadic attention, pupil light response, vertical meridian, visual field asymmetry

## Abstract

Vision is introspectively stable, yet every eye movement moves the image of the world on the retina. The dominant view states that attention must precede saccades to prepare the brain for the postsaccadic retinal input, which ensures a stable visual experience. A recent surge of studies investigated visual asymmetries around the visual field, including asymmetries in presaccadic attention. Such studies demonstrated benefits of presaccadic attention on task performance for horizontal and downward saccades, but strikingly no such benefit was observed for upward saccades. An absence of upward presaccadic shifts would contrast the dominant view and indicate that presaccadic attention may not be necessary to ensure perceptual continuity. Here, we capitalized on the fact that the pupil light response robustly tracks spatial attention to investigate whether presaccadic attention shifts up‐ and downwards. We manipulated whether the landing brightness of the ensuing saccade could be prepared for prior to the saccade. Specifically, we either presented brightness patches throughout the trial or only presented these upon saccade onset. In two experiments, we observed earlier pupil light responses for both up‐ and downward saccades when the landing brightness could be prepared for presaccadically. This shows that presaccadic attention shifted prior to up‐ and downward saccades and agrees with presaccadic attention being instrumental in realizing a stable visual experience. Reconciling previously contradictory findings, presaccadic attention can be shifted without necessarily yielding perceptual benefits for all facets of visual processing at the attended location. Nevertheless, our findings demonstrate presaccadic attention to shift along the vertical meridian.

## Abstract 2

Dominant theories posit that attentional shifts prior to saccades enable a stable visual experience despite abrupt changes in visual input caused by saccades. However, recent work may challenge this view as no presaccadic benefit was observed when preparing upward saccades. Here, we capitalized on the fact that the pupil light response tracks spatial attention physiologically, and demonstrated presaccadic attention to shift up‐ and downwards. Thus, presaccadic attention may facilitate our continuous and stable visual experience.

## 1 Introduction

Humans and other foveal animals make fast, jerk‐like eye movements called saccades to inspect objects in the environment at a high visual acuity (Findlay and Gilchrist 2003). Saccades are executed 3–4 times per second (Henderson 2003; Henderson and Hollingworth 1998), and each saccade abruptly moves the image of the world on the retina (Cavanagh et al. 2010; Melcher 2007; O'Regan 1992). In the words of O'Regan (1992, 463), “the real mystery” of visual perception is that it is introspectively continuous and stable despite the highly frequent, abrupt and substantial changes in retinal input caused by eye movements. How does the brain solve this mystery of realizing a seamless visual experience?

The dominant view posits that presaccadic shifts of attention are instrumental for our seamless visual experience (Cavanagh et al. 2010; Currie et al. 2000; Deubel and Schneider 1994; Deubel et al. 1998; Mathôt and Theeuwes 2011; McConkie and Currie 1996; Rolfs 2015). Prior to each saccadic eye movement, attention already moves toward the saccade target before the eye starts moving (Deubel and Schneider 1996; Kowler et al. 1995). Presaccadic shifts of attention alter visual perception at the location of the saccade target in a number of ways, including enhanced contrast sensitivity, orientation tuning, and differential processing of spatial frequency (Hanning et al. 2022, 2024; Kroell and Rolfs 2021; Kwak et al. 2024; Kwak et al. 2023; Li et al. 2019; Ohl et al. 2017; Rolfs and Carrasco 2012), rendering the upcoming saccade target more “fovea‐like” (Carrasco 2018; Li et al. 2016; Rolfs 2015). In addition to enhancing perception presaccadically, the saccade target is encoded into working memory (Schut et al. 2017; Tas et al. 2016). In turn, this memory representation of the saccade target allows to bridge perception from one fixation to another (Van der Stigchel and Hollingworth 2018). Thus, presaccadic attention is dominantly posited as a key mechanism in realizing a seamless visual experience despite moving the image of the world on the retina with each saccade.

Two recent studies may challenge this dominant view, as presaccadic attention may not always precede an eye movement. Hanning et al. (2022, 2024) investigated the effect of presaccadic attention on contrast sensitivity across directions. To this end, participants performed an orientation discrimination task while the contrast of the target stimulus was titrated (using a staircase procedure). As expected, contrast sensitivity was enhanced when preparing saccades toward the target stimulus in left, right, and downward directions. In contrast, presaccadic attention did not enhance contrast sensitivity when preparing upward saccades. This striking pattern was observed in both studies. The lack of perceptual benefits when preparing upward saccades may suggest that presaccadic attention is not shifted prior to upward saccades. However, executing upward saccades does not break the introspectively continuous visual experience. If presaccadic attention shifts do not occur for upward saccades, it is possible that presaccadic shifts are not mandatory to effectuate a continuous and stable visual experience, as claimed by dominant theories (Cavanagh et al. 2010; Currie et al. 2000; Deubel and Schneider 1994; Deubel et al. 1998; Mathôt and Theeuwes 2011; McConkie and Currie 1996; Rolfs 2015).

To directly address this possibility, we here investigated whether presaccadic attention is shifted for up‐ and downward saccades without the need for an overt manual response by using pupillometry. The pupil light response (PLR) entails constrictions to bright and dilations to dark visual input (Loewenfeld 1958). The PLR is not merely a reflex but strongly modulated by attention (Binda and Murray 2015; Koevoet et al. 2024a; Mathôt and Van der Stigchel 2015; Strauch, Wang, et al. 2022). Deploying covert attention toward a dark or bright stimulus, respectively, constricts and dilates the pupil even when the overall brightness is kept constant (Binda et al. 2014; Haab 1886; Mathôt et al. 2013, 2014; Naber et al. 2013). This phenomenon has also been extended to shifts of presaccadic attention: Mathôt et al. (2015) found that when the brightness of the upcoming saccade target could be prepared prior to a saccade, the PLR emerged substantially earlier. This demonstrates that presaccadic attention prepares the perception of saccade targets and makes the PLR a promising tool for the current purpose (also see Wang and Munoz 2018).

We here employed an adapted version of the paradigm used in Mathôt et al. (2015). Crucially, this allowed us to manipulate whether the landing brightness of the saccade target could be prepared presaccadically or not. We did so by either presenting the landing brightness throughout the entire trial (i.e., able to prepare for the landing brightness) or by only presenting the landing brightness upon saccade onset (i.e., not able to prepare for the landing brightness). Although detection/discrimination tasks have been instrumental in studying attention, they have some limitations. One relevant limitation is that detection/discrimination tasks alone cannot discern between possible explanations for null‐results. Specifically, detection/discrimination tasks cannot discern between situations wherein attention was not shifted, or that attention did shift but did not enhance task performance. By contrast, our method circumvents this issue by allowing to track (presaccadic) attention in isolation of any detection/discrimination task and their accompanying cognitive/motor factors (e.g., decision‐making related processes or preparing manual responses).

To foreshadow our results from two experiments, when presaccadic preparation of the upcoming landing brightness was possible, the PLR emerged earlier. Our data demonstrated that presaccadic attention shifts both up‐ and downward. These results reconcile the aforementioned contradictory findings and suggest that presaccadic attention plays an instrumental role in ensuring a continuous and stable visual experience.

## 2 Methods

All analyses scripts and data are available via the Open Science Framework: https://osf.io/ckp3r/. None of the experiments were preregistered.

### 2.1 Participants and Power Analysis

Thirteen participants with normal or corrected‐to‐normal vision took part in Experiment 1. Participants reported no history or diagnosis of attention‐deficit (hyperactivity) disorder or autism spectrum disorder. One participant did not complete the full session and was excluded from the data analysis. Data from twelve participants (including D.K.; Age: *M* = 21.42, range = [19–26], 9 females, 3 males, all right‐handed) were analyzed.

The sample size was based on previous work (Hanning et al. 2022, 2024; Mathôt et al. 2015). We additionally ran a power analysis using G*Power (v3.1.9.6) for a repeated‐measures ANOVA with an alpha of 0.05, power of 0.99, and an effect size of *η*_*p*_^2^ = 0.730—based on the effect size reported in Mathôt et al. (2015) when contrasting the PLR onset between constant and onset conditions (see Procedure). Although the power analysis indicated that only four participants were necessary to achieve adequate power, we set our target sample to 12 to stay consistent with Hanning et al. (2022, 2024). The experimental procedure was approved by the local ethical review board (approval code: 21‐0297).

### 2.2 Apparatus

Gaze position and pupil size of the right eye were recorded at 1000 Hz with an EyeLink 1000+ (SR Research, Mississauga, Ontario, Canada). Participants were seated approximately 67.5 cm away from the monitor (2560 × 1440; 100 Hz) in a chin‐ and headrest. Stimuli were presented using PsychoPy (v2021.2.3; Peirce et al. 2019).

### 2.3 Procedure

**FIGURE 1:**
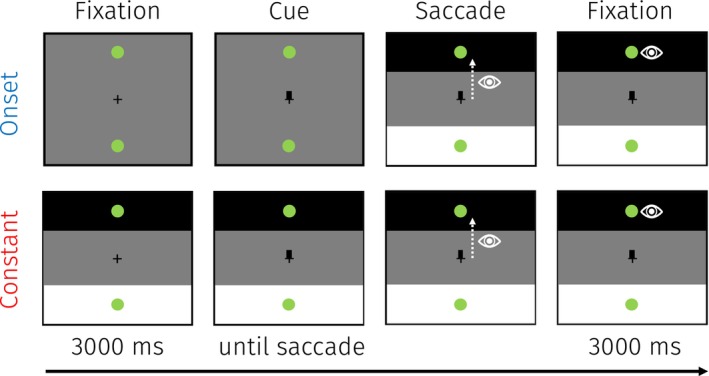
Trial overview for Experiment 1. Participants fixated the central fixation cross until cued to saccade toward the upper or lower green dot as fast as possible. Participants maintained their gaze on the green saccade target for 3 s to allow for a PLR to emerge. Participants could presaccadically prepare for the landing brightness in the constant condition, but not in the onset condition because here the landing brightness only appeared upon saccade execution. This example represents a trial with an upward saccade and a dark landing brightness.

Participants performed a cued‐saccade task (Figure 1). Each trial started with a fixation period (3 s) during which two potential green saccade targets (192 cd/m^2^; radius 0.25°) were presented 10° above and below the central fixation cross. Participants were then cued toward the upper or lower saccade target by respectively making the upper or lower part of the fixation cross thicker. Participants were instructed to execute a saccade toward the cued target as fast as possible and to subsequently keep fixating the target (3 s) to allow for the PLR to emerge. A 1 s intertrial interval was implemented so participants could blink and to let the pupil's size return to baseline.

The upper and lower visual fields were always tagged with one bright (179 cd/m^2^; 10° vertically centered on the saccade target) and one dark (0.32 cd/m^2^) patch that covered the entire width of the display to elicit robust PLRs (based on Mathôt et al. 2015; Strauch, Romein, et al. 2022). Each brightness patch appeared in the upper and lower visual fields equally often. In constant trials, the brightness patches were presented throughout the trial, making potential presaccadic preparation of the landing brightness possible. In onset trials, the brightness patches were presented only upon saccade onset so that the ensuing landing brightness could not be prepared for presaccadically. We detected saccade onsets during the task whenever the eye moved more than 3° from the vertical center. This manipulation allowed us to investigate whether presaccadic attention was shifted up and downward by employing the PLR.

Participants completed a total of 320 trials equally distributed across the eight conditions (2 constant/onset × 2 up/down × 2 dark/bright landing brightness). Every 40 trials, participants took a break. The order of trials was fully randomized (i.e., mixed design), and participants completed eight practice trials at the start of the experiment.

### 2.4 Data Processing and Analysis

Data processing and analyses were performed using custom Python scripts, and were based on previous work (Koevoet et al. 2023; Mathôt and Vilotijević 2022; Mathôt et al. 2015; Strauch, Wang, et al. 2022). Gaze position and pupil size were downsampled to 100 Hz. Blinks were reconstructed using the blinkreconstruct function (in ‘advanced’ mode) from the DataMatrix package. This blink reconstruction algorithm is described in detail in Mathôt and Vilotijević (2022). Briefly, the recursive algorithm first identifies blinks based on velocity changes in pupil size (5 arbitrary units [from the Eyelink] per ms). Then, missing data is interpolated using a cubic spline interpolation. If cubic spline interpolation is not possible (i.e., not enough usable data points around the blink), linear interpolation is performed. If linear interpolation also cannot be reliably performed (i.e., more than 500 ms of invalid/missing data), data is left as invalid/missing. After identifying (and if possible reconstructing) a blink, the recursive algorithm starts again from scratch, and continues to restart until no new blinks are identified.

Following Mathôt et al. (2015), pupil size data were locked to saccade onset. Baseline correction was applied by subtracting the median pupil size from the first 100 ms prior to saccade onset. Saccade onsets were detected using a velocity threshold of 50°/s (Koevoet et al. 2025). Trials with saccade onsets occurring earlier than 100 ms or later than 1000 ms after cue onset were discarded (Mathôt et al. 2015). Additionally, trials where the online saccade detection occurred before saccade onset as determined offline were discarded because the landing brightness could have appeared prior to saccade onset in these trials. A total of 3642 trials (94.84%) were analyzed in Experiment 1.

We used linear mixed‐effects models as an initial way to determine when dark and bright landing positions started to differ in the different conditions. We analyzed each sample over time (every 10 ms; Wilkinson Notation: pupil size ∼ landing brightness + (1 + landing brightness|participant)). We modeled intercepts and slopes for each participant to control Type 1 errors (Barr 2013; Barr et al. 2013). As we were primarily interested in latency differences in the PLR between conditions, separate models were conducted for each of the four conditions (2 constant/onset × 2 up/down) (similar to Mathôt et al. 2015). Throughout the study, we set *α* = 0.05, which corresponds to *t* > 1.96 for the linear mixed‐effects models.

To directly test the differences in PLR latency between conditions, we additionally modeled the PLR using an exponential decay function (adapted from Hoeks and Levelt 1993). Although many functions fit the PLR adequately (Cai et al. 2023; Korn and Bach 2016), we opted for this approach as it provides straightforward outcome measures and has been used in previous work investigating presaccadic attention using pupil size (Mathôt et al. 2015). We calculated the average difference between dark and bright landing brightness for each condition across time and fitted the model to each participant separately. The exponential decay function returns four parameters: the start and end values, the slope of the response, and the onset of the PLR. The start value refers to the extent of the PLR at the start of the difference trace (i.e., the difference between dark and bright trials at saccade onset). The end value indicates the extent of the PLR at the end of the modeled function. The slope indicates the steepness of the modeled curve. The onset parameter indicates when the curve starts to decrease and therefore reflects when the PLR emerges. To assess the fits of the model, we computed Pearson correlations for every fitted line and the raw trace. The exponential decay function fit the data well (*r* > 0.98 for all participants). Repeated‐measures two‐way ANOVAs were used to analyze model parameters across onset/constant and direction conditions using JASP (v0.18.1). We here report the outcomes of the analyses of the start values and PLR onset latencies as these are most relevant for the current purpose—analyses of the other parameters, as well as point‐by‐point comparisons of PLR difference traces are provided in the Supporting Information.

## 3 Results

We first analyzed saccade latencies with a linear mixed‐effects model, Wilkinson Notation: Saccade latency ∼ Constant/Onset × Direction + (1 + Direction|Participant). Saccade latencies were numerically but not significantly shorter for up‐ compared with downward saccades (*M*_up_ = 359.21 ms, *M*_down_ = 369.40 ms, *β* = 9.412, SE = 5.778, *t* = 1.629, *p* = 0.103). The other effects were also not significant (*t*s < 1.2, *p*s > 0.25).

**FIGURE 2:**
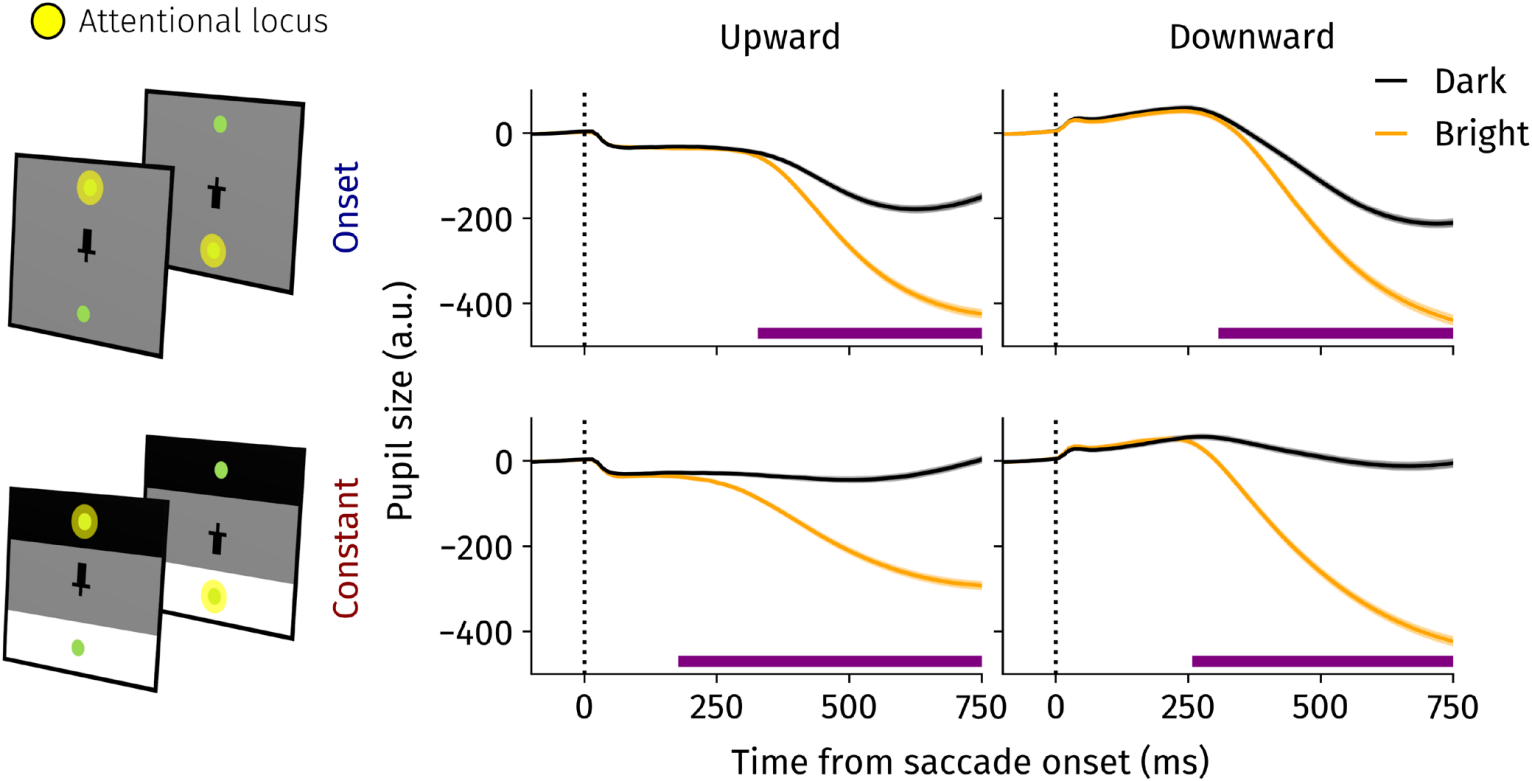
Pupil size locked to saccade onset for the different direction and constant/onset conditions in Experiment 1. Landing brightness differences became significant earlier for the constant conditions. Horizontal purple line indicates significant differences between the dark and bright landing brightness, *p* < 0.05. Error bands indicate standard errors of the mean.

We investigated whether presaccadic shifts of attention move up‐ and downwards. We capitalized on the fact that presaccadic attention makes the PLR emerge earlier by preparing for the ensuing landing brightness (Mathôt et al. 2015). Presaccadic shifts of attention should therefore result in earlier onsets of the PLR whenever a brightness could be prepared prior to saccade onset. Linear mixed‐effects models were fit over time split across constant and onset conditions and saccade directions to investigate this possibility (Figure 2).

For upward saccades, pupil size differed significantly between landing brightness conditions 330 ms following saccade onset in onset trials and already 180 ms in the constant condition. A similar pattern emerged for downward saccades: in the onset and constant conditions, a significant pupil light response was observed 310 ms and 260 ms after saccade onset, respectively. In other words, for both up‐ and downward‐directed saccades, the PLR emerged earlier when the brightness could be prepared for presaccadically. This is a first indication that attention is shifted prior to up‐ and downward saccades.

As expected, we found small decreases and increases in pupil size shortly after up‐ and downward saccades, respectively. This difference is driven by a change in angle between the eye and the camera of the eye tracker, also known as the pupil foreshortening error (Hayes and Petrov 2016). As we were primarily interested in differences between dark and bright landing trials, we could account for the pupil foreshortening error effectively, and this did not affect our results.

**FIGURE 3:**
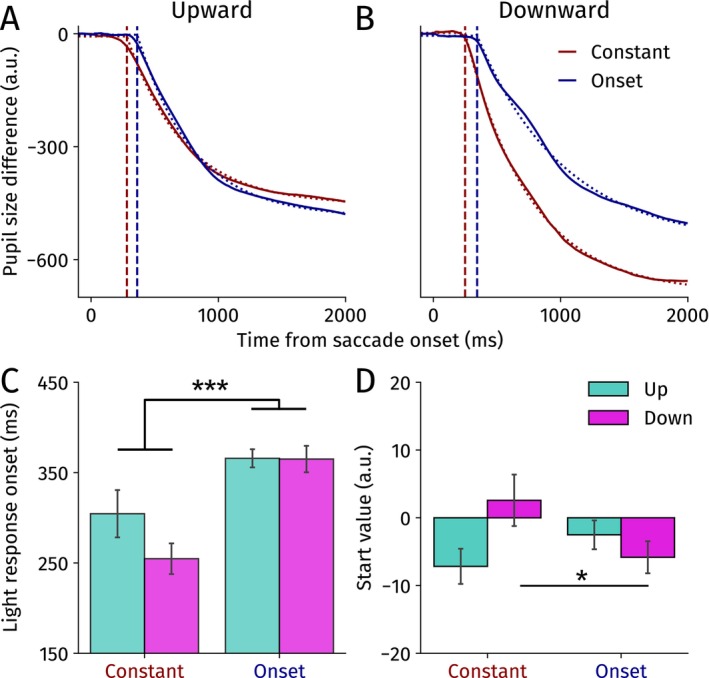
Average model fits for (A) upward and (B) downward saccades for the constant and onset conditions separately for Experiment 1. Thick lines show the averaged differences traces, and dashed traces show the modeled pupil light response. Vertical dashed lines indicate the pupillary light response onset latencies. Horizontal dashed lines reflect start values. (C) Modeled pupillary light response onset latencies (relative to saccade onset) for the constant and onset conditions split per saccade direction. (D) Modeled difference start values upon saccade onset. Error bars yield standard errors of the mean. *p < 0.05, ***p < 0.001.

To statistically test for a latency effect directly, we fit an exponential decay function to the difference between bright and dark trials for the constant and onset conditions split per saccade direction separately (Figure 3A,B; see Methods for details). We specifically focused on the onset latency of the PLR and the start value parameters as they are affected by presaccadic attention most decisively (see Supporting Informations for the other parameters).

A repeated measures ANOVA showed that the PLR emerged significantly earlier in the constant than in the onset condition, *F*(1,11) = 22.91, *p* < 0.001, *η*_*p*_^2^ = 0.676 (Figure 3C). There was no significant main effect of direction *F*(1,11) = 2.22, *p* = 0.17, *η*_*p*_^2^ = 0.168, nor an interaction effect, *F*(1,11) = 2.73, *p* = 0.13, *η*_*p*_^2^ = 0.199. This complements our analysis of pupil size over time (Figure 2) and together demonstrates that the onset latency of the PLR for both up‐ and downward saccades is decreased when the ensuing landing brightness could be prepared for presaccadically. Our findings reveal that presaccadic attention shifts upwards, which indeed fits the dominant view that presaccadic shifts facilitate perceptual stability.

One possible caveat is that, despite our baseline correction, start values slightly differed between conditions (Figure 3D; horizontal dashed lines at the start of Figure 3A,B). Specifically, we observed an interaction effect between saccade direction and preparation condition, *F*(1,11) = 6.76, *p* = 0.025, *η*_*p*_^2^ = 0.381. Start values were significantly lower in the constant than in the onset condition for downward saccades, *t*(11) = 2.65, *p* = 0.023, Cohen's *d* = 0.76, and no significant difference was observed for upward saccades, *t*(11) = 1.35, *p* = 0.20, Cohen's *d* = 0.39 (although numerically the opposite pattern was found). The other effects were not significant (constant vs. onset: *F*(1,11) = 0.75, *p* = 0.40, *η*_*p*_^2^ = 0.064; up vs. down: *F*(1,11) = 0.78, *p* = 0.40, *η*_*p*_^2^ = 0.066).

What caused this interaction effect in start values? The PLR shows a vertical asymmetry: the pupil responds stronger to brightness in the upper compared to the lower vertical meridian (see Supporting Information; Cai et al. 2023; Kardon et al. 1991; Strauch, Romein, et al. 2022). In constant trials, this asymmetry led to differences in pupil size before cue onset due to the presentation of different brightnesses in the upper and lower visual fields. Despite applying baseline correction, we could not fully eliminate the asymmetry effect in the current data. Although unlikely from visual inspection (Figure 2), it is possible that these differences in start value affected our PLR onset latency estimates. This unlikely scenario was ruled out in Experiment 2.

## 4 Experiment 2

The purpose of Experiment 2 was twofold. First, we aimed to replicate the results to test the robustness of our finding while controlling for differences in brightness between the upper and lower visual field. Second, we slightly adjusted the design to make the findings more directly comparable with previous reports that found no beneficial effects of presaccadic attention for upward saccades (Hanning et al. 2022, 2024).

## 5 Methods

The methods and analyses for Experiment 2 were identical to Experiment 1 unless specified explicitly.

### 5.1 Participants

Fourteen participants with normal or corrected‐to‐normal vision took part in Experiment 2. Data from two participants were excluded due to excessive blinking and noisy model fits^1^. Data from 12 participants (including D.K.; Age: *M* = 25.17, range = [22–29], 6 females, 1 left‐handed) were included in the final analyses.

### 5.2 Cued Saccade Task and Procedure

**FIGURE 4:**
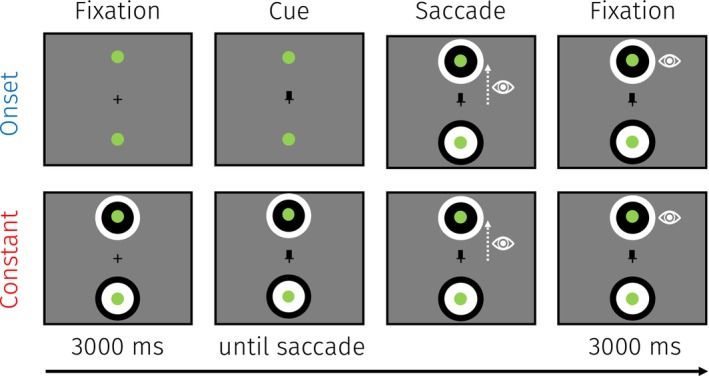
Trial overview for Experiment 2. Participants fixated the central fixation cross, until cued to saccade toward the upper or lower green dot as fast as possible. Participants maintained their gaze on the green saccade target for 3 s to allow for a PLR. Participants could presaccadically prepare for the landing brightness in the constant condition, but not in the onset condition since here the landing brightness only appeared upon saccade execution. This example represents a trial with an upward saccade and a dark landing brightness.

The task and procedure were identical to the one employed in Experiment 1 besides the following changes (Figure 4). To reduce the effects of the vertical asymmetry on the PLR, we used bright and dark circular stimuli. The inner (radius 2.83°) and outer (radius 4°) brightnesses had the same surface area. These stimuli allowed us to present both dark and bright stimuli in the upper and lower parts of the visual field on each trial—this effectively equates the brightness in the upper and lower visual fields. We also increased the inter‐trial interval to decrease the chance of observing differences in start values (1 s vs. 2 s). Due to the longer trial duration, we decreased the total number of trials to 30 per condition for practical reasons and to limit fatigue. Additionally, we controlled for possible effects of the eyes' position with respect to the monitor (i.e., the eyes could be closer to the upper or lower saccade target) by aligning participants' eyes to the vertical center of the monitor. Lastly, the green saccade targets were now positioned at an eccentricity of 8° (as opposed to 10° in Experiment 1) to be more comparable with previous studies (Hanning et al. 2022, 2024).

Again the exponential decay function fit the PLR well (*r*: *M* = 0.98, range = [0.72–0.99]). After data exclusion, 2832 trials (98.33%) remained for statistical analyses.

## 6 Results

As in Experiment 1, we first analyzed saccade latencies. Up‐ compared with downward saccades had shorter saccade latencies (*M*_up_ = 328.93 ms, *M*_down_ = 350.35 ms, *β* = 21.893, SE = 8.059, *t* = 2.716, *p* = 0.007), in line with previous work (Hanning et al. 2022, 2024). The other effects were not significant (*t*s < 0.69, *p*s > 0.49).

**FIGURE 5:**
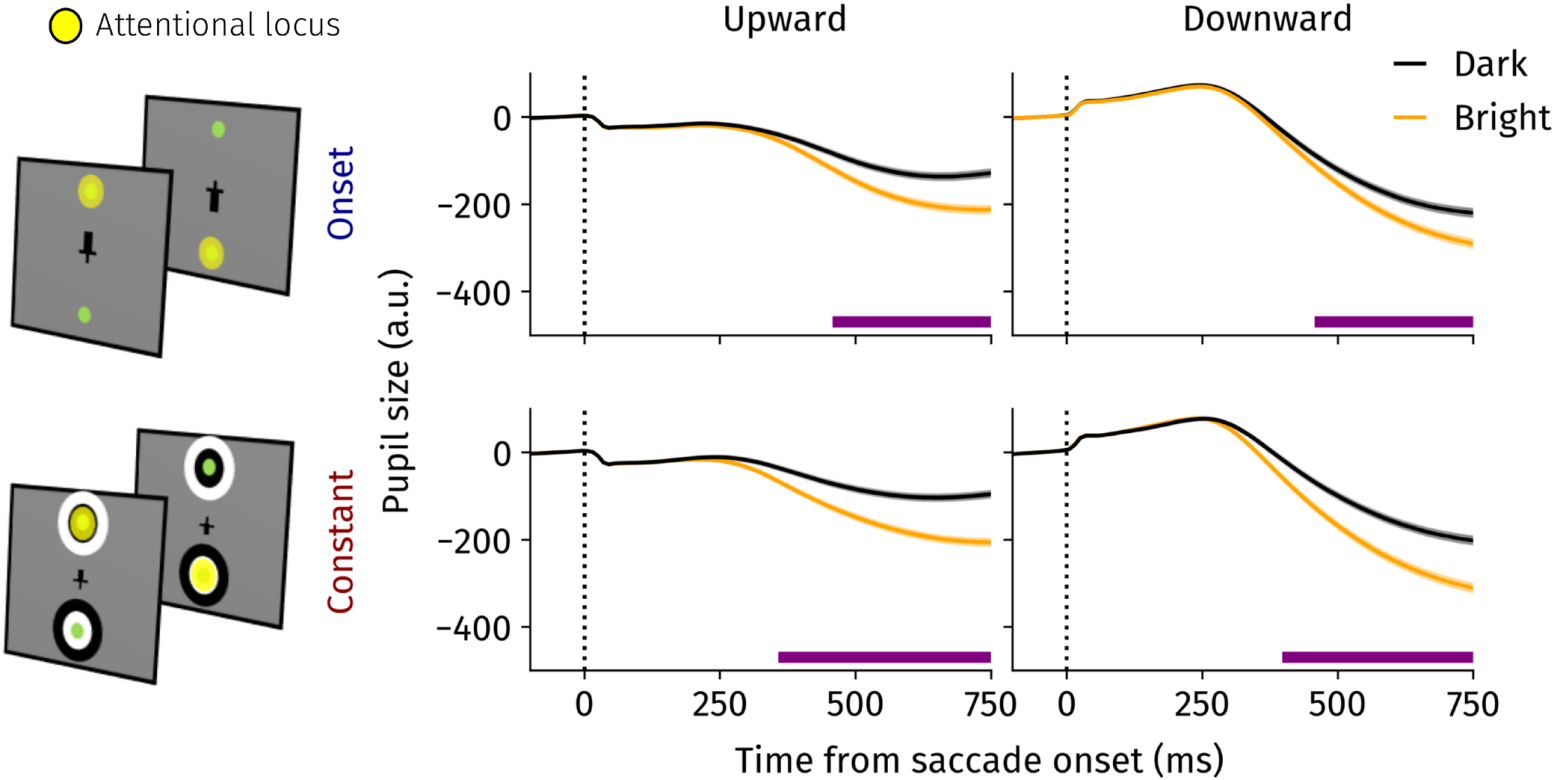
Pupil size locked to saccade onset for the different direction and constant/onset conditions in Experiment 2. Landing brightness differences became significant earlier for the constant conditions. Horizontal purple line indicates significant differences between the dark and bright landing brightness, *p* < 0.05. Error bands indicate standard errors of the mean.

We then analyzed the PLR over time separately for the constant and onset conditions split per saccade direction (Figure 5). Differences emerged earlier in the constant (360 ms) compared to the onset condition for upward saccades (460 ms), and the same pattern was observed for downward saccades (400 vs. 460 ms following saccade onset). This replicates our findings from Experiment 1 and again tentatively suggests a presaccadic shift of attention in both directions.

**FIGURE 6:**
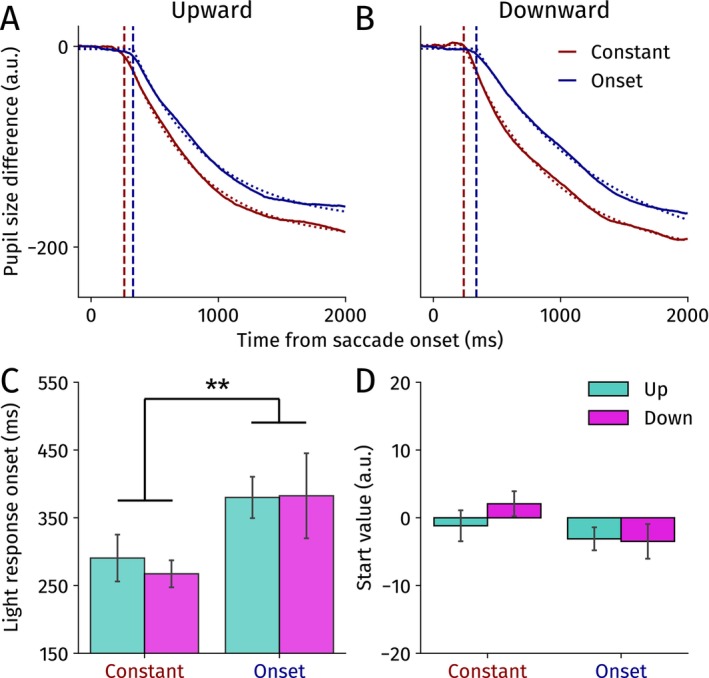
Average model fits for (A) upward and (B) downward saccades for the constant and onset conditions separately for Experiment 2. Thick lines show the averaged differences traces, and dashed traces show the modeled pupil light response. Vertical dashed lines indicate the pupillary light response onset latencies. Horizontal dashed lines reflect start values. (C) Modeled pupillary light response onset latencies (relative to saccade onset) for the constant and onset conditions split per saccade direction. (D) Modeled difference start values upon saccade onset. Error bars yield standard errors of the mean. **p < 0.01.

We fit exponential decay functions to the PLR difference traces (Figure 6). In line with the analyses over time, a repeated‐measures ANOVA showed that the PLR emerged earlier in the constant than in the onset condition, *F*(1,11) = 10.44, *p* = 0.008, *η*_*p*_^2^ = 0.49. The main effect of direction and the interaction effect were not significant, *F*s(1,11) < 0.10, *p*s > 0.77, *η*_*p*_^2^s < 0.008. This replicates the key finding from Experiment 1: when the landing brightness could be prepared presaccadically, the PLR emerged earlier, both for up‐ and downward saccades. Together, this shows that attention is shifted presaccadically along the vertical meridian.

In Experiment 1, we found an interaction effect between direction and preparation in PLR difference start values. Using different stimuli and longer intertrial intervals, we aimed to diminish this effect to ensure this did not drive the results from Experiment 1 (Note that we show that the vertical PLR asymmetry has been ameliorated effectively during the pre‐cue fixation period in the Supporting Information). A repeated‐measures ANOVA showed no significant main effects, *F*s(1,11) < 3.90, *p*s > 0.07, *η*_*p*_^2^s < 0.27, nor a significant interaction effect in PLR difference start values, *F*(1,11) = 0.74, *p* = 0.41, *η*_*p*_^2^ = 0.06. This demonstrates that the possibility to prepare the landing brightness presaccadically leads to an earlier PLR onset and that this effect is not driven by differences in start values.

To assess the robustness of our results, we also analyzed the data while locking the pupil data to saccade offset. This analysis, in combination with analyses of saccadic landing precision, provides converging evidence that presaccadic attention is shifted up‐ and downward (see Supporting Information).

## 7 Discussion

Dominant theories state presaccadic attention to be instrumental in facilitating a continuous and stable visual experience (Cavanagh et al. 2010; Currie et al. 2000; Deubel and Schneider 1994; Deubel et al. 1998; Mathôt and Theeuwes 2011; McConkie and Currie 1996; Rolfs 2015). Two recent studies may challenge this proposition as presaccadic attention did not boost perceptual performance prior to upward saccades specifically (Hanning et al. 2022, 2024). We here addressed whether presaccadic attention is shifted prior to upward saccades. To study this, we capitalized on the fact that the PLR is strongly modulated by attention and does not necessitate an overt manual response (Binda et al. 2014; Koevoet et al. 2024b; Mathôt and Van der Stigchel 2015; Mathôt et al. 2013, 2014; Naber et al. 2013; Strauch, Wang, et al. 2022). Across two experiments, we found the PLR to emerge earlier when the ensuing landing brightness could be prepared for prior to up‐ and downward saccades. This clearly demonstrates that presaccadic attention is shifted prior to upward saccades, which is in line with the dominant view that presaccadic attention facilitates visual stability.

How can the current findings be reconciled with Hanning et al. (2022, 2024)? Hanning et al. (2022, 2024) found a striking lack of presaccadic attentional benefit on contrast sensitivity when preparing upward saccades. However, Kwak et al. (2023) found a boost in visual acuity when preparing saccades in all cardinal directions, including upward saccades. In addition, presaccadic attention appears to reshape the contrast sensitivity function differently for horizontal than for vertical saccades (Kwak et al. 2024). These findings indicate that presaccadic attention alters distinct facets of visual processing differently across directions. Together, the effects of presaccadic attention on visual processing may differ based on where it is deployed (Hanning et al. 2022, 2024; Himmelberg et al. 2023; Kwak et al. 2024; Liu et al. 2024), yet our data indicate that presaccadic attention is deployed at the intended saccade target. Put differently, this indicates that presaccadic attention can be shifted without necessarily altering (or boosting) behavioral performance at the saccade target. From this, behavioral performance on perceptual tasks may therefore not always accurately indicate whether attention was shifted toward a target. Our employed PLR‐based method circumvents such issues by physiologically assessing whether presaccadic attention was shifted without necessitating a discrimination/detection task with overt manual responses.

Visual field asymmetries in early visual cortex could explain why presaccadic attention does not affect perception equally across the visual field. Visual perception is best along the horizontal meridian and more detailed at the lower than the upper visual field (reviewed in Himmelberg et al. 2023). Visual asymmetries are also apparent in early visual cortex (Silva et al. 2018) and correlate with perception (Himmelberg et al. 2022). In the context of presaccadic attention, Hanning et al. (2024) found a correlation between V1 surface area representing the upward saccade target and the extent of presaccadic enhancement (or decrement) of contrast sensitivity across participants. This provides a first step in understanding why presaccadic attention may affect perception differently across the visual field. Future work could explore whether such relationships between V1 surface and perception generalize to other forms of attention such as exogenous or endogenous covert shifts.

One notable difference between our current experiments and the studies by Hanning et al. (2022, 2024) is that the total number of targets differed. The current experiments only had two possible saccade targets compared with the four targets employed by Hanning et al. (2022, 2024). One may argue that this affected the strategies employed by participants during the task. Although we cannot fully rule this out, we are confident that participants only started saccade programming upon cue onset. To support this, in Experiment 2 (where we controlled for the vertical asymmetries in PLR) we did not find any systematic modulations of the PLR in constant trials prior to cue onset (see Supporting Informations). Therefore, as saccade programming occurred upon cue onset, we deem it likely that similar strategies were employed across studies, but this remains to be tested directly.

We have recently demonstrated that the intrinsic costs of planning and executing saccades are quantifiable using pupil size (Koevoet et al. 2023). Although subtle, saccade costs as measured with pupil size robustly predict where one decides to look next (Koevoet et al. 2025; Koevoet et al. 2024c)—which is in line with previous work (Hoppe and Rothkopf 2016; Kadner et al. 2022; Shadmehr et al. 2019; Thomas et al. 2022). Saccade costs have been shown to differ across directions (Koevoet, Van Zantwijk, et al. 2024; Koevoet et al. 2023). Specifically, the pupil dilates more prior to the execution of down‐ compared with upward saccades (Koevoet et al. 2024c). One possibility for this may be that contrast sensitivity is not enhanced in a similar way when preparing upward saccades. Other factors such as saccade latency and landing precision also differ between up‐ and downward saccades, which may also play a role (e.g., Greenwood et al. 2017; Hanning et al. 2022, 2024). However, the relationships between saccade cost, saccade metrics, cortical, perceptual, and attentional asymmetries remain unknown and constitute an exciting open avenue for future work.

Most of the current findings are in line with a previous study investigating presaccadic attention along the horizontal meridian using the PLR (Mathôt et al. 2015). Here, we replicate and extend the core finding from the previous study: When the ensuing landing brightness could be prepared presaccadically, the PLR emerged approximately 85 ms and 95 ms earlier in Experiments 1 and 2, respectively (similar to Mathôt et al. 2015). This dovetails with previous studies revealing that presaccadic attention exerts its perceptual effects approximately 100 ms prior to saccade onset (Deubel 2008; Li et al. 2016; Ohl et al. 2017; Rolfs and Carrasco 2012). We extend the previous work by showing that presaccadic attentional effects on the PLR generalize to the vertical meridian. Moreover, we introduced new stimuli in Experiment 2 that show that presaccadic attention is deployed focally. Specifically, the PLR was tuned to the brightness of the inner and not the outer circle, implying that presaccadic attention is deployed focally around the saccade target. This approach could be adapted to systematically study the focality/breadth of attentional deployment.

One difference between the current findings and Mathôt et al. (2015) was that we did not observe a preparatory PLR effect prior to saccade onset. We propose two reasons for this discrepancy. First, we used a visual cue to indicate the saccade target, whereas the original study used verbal auditory cues (i.e., left vs. right). These verbal auditory cues led to relatively slow saccade onsets (∼540 ms) causing longer cue‐saccade intervals than in the current experiment. Since the pupil response is relatively sluggish, a longer cue‐saccade interval increases the chance of observing attentional modulations of the PLR prior to saccade onset. Second, we observed a substantial vertical asymmetry in the PLR in Experiment 1. Specifically, the PLR reflected the brightness of the upper field more strongly than the lower visual field even prior to cue onset. This asymmetry could have masked a preparatory PLR effect preceding saccade onset. Although a similar pattern can be observed for preferential processing of the left over the right visual field (i.e., pseudoneglect), these effects are much smaller in size (Strauch, Romein, et al. 2022; Ten Brink et al. 2023), ultimately making it less likely that these effects masked attentional preparation effects.

Can the observed postsaccadic pupillary response be ascribed to presaccadic attention? Based on at least three points, we are confident that the evoked PLRs in our experiments reflect presaccadic attention. First, the PLR is known to be modulated strongly by the allocation of covert attention (Binda et al. 2014; Haab 1886; Mathôt and Van der Stigchel 2015; Naber et al. 2013). Given the tight relationship between eye movements and attention (Awh et al. 2006; Rizzolatti et al. 1987), it is likely that the PLR is also sensitive to presaccadic attention (also see Mathôt et al. 2015). Second, our main manipulation between the constant and onset conditions is solely based on what is presented before saccade initiation. Therefore, differences in the postsaccadic pupillary response between these conditions must be attributed to processes unfolding prior to saccade execution. Third, the pupillary response is sluggish and lags behind real‐time cognitive processing (e.g., Beatty 1982; Strauch, Wang, et al. 2022). Therefore, the pupil cannot resolve the presaccadic attentional process prior to saccade execution, but instead emerges postsaccadically. Thus, the PLR closely tracked presaccadic attention in the current tasks.

Together, our results clearly demonstrate that presaccadic attention is deployed prior to up‐ and downward saccades. This supports the dominant view that presaccadic attention is instrumental in effectuating a stable visual experience. To reconcile the current findings with studies reporting a lack of perceptual benefits when presaccadic attention is shifted upwards, we show that presaccadic attention *is shifted upwards* but note that it may affect processing there differently compared with other directions (Hanning et al. 2022, 2024; Kwak et al. 2024). More work is necessary to directly test this proposition as well as to identify possible neural correlates of these processes. One promising avenue may be to combine the currently employed PLR method with psychophysical tasks.

## Author Contributions

**Damian Koevoet:** conceptualization, data curation, formal analysis, investigation, methodology, software, visualization, writing – original draft, writing – review and editing. **Marnix Naber:** conceptualization, methodology, supervision, writing – review and editing. **Christoph Strauch:** conceptualization, supervision, writing – review and editing. **Stefan Van der Stigchel:** conceptualization, funding acquisition, supervision, writing – review and editing.

## Conflicts of Interest

The authors declare no conflicts of interest.

## Supporting information


Data S1.

## Data Availability

Analyses, scripts, and data are available via the Open Science Framework: https://osf.io/ckp3r/.

## Endnotes

^1^
Note that including all participants in the analyses results in conceptually identical findings.
