# Supplementary material for: Presaccadic Attention Shifts Up‐ and Downwards: Evidence From the Pupil Light Response
Source: Psychophysiology. 2025 Mar 17;62(3):e70047. doi: 10.1111/psyp.70047 (PMC11913767; doi:10.1111/psyp.70047)
Supplement: Supplementary file 1 — Data S1. [file PSYP-62-e70047-s001.docx]

Supplementary Material to *Presaccadic Attention Shifts Up- and Downwards: Evidence from the Pupil Light Response*

## Damian Koevoet*, Marnix Naber, Christoph Strauch, and Stefan Van der Stigchel

Experimental Psychology, Helmholtz Institute, Utrecht University, The Netherlands

*Correspondence to [d.koevoet@uu.nl](mailto:d.koevoet@uu.nl)

# Slope and Stop Value Analyses

In the main manuscript, the pupil light response (PLR) was fit using an exponential decay function (Hoeks & Levelt, [1993](#_bookmark6); Mathôt et al., [2015](#_bookmark9)). This function returns four parameters: start value, PLR onset latency, slope and stop value. Analyses of the start value and PLR onset latency are described in the main manuscript. We will detail the outcomes from the slope and stop value analyses here.

For each outcome measure, we conducted a repeated-measures ANOVA (2 on- set/constant x 2 up/down) for each experiment separately. For the slope analy- ses in Experiment 1, we found a main effect of saccade direction, a main effect of constant/onset condition and a marginal interaction effect, *F*s(1,11) > 4.5, *p*s < .058, *η*^2^s > .29. However, none of these effects replicated in Experiment 2, *F*s(1,11) < 1.95, *p*s > .20, *η*^2^s < .16.

*p*

*p*

We also observed an inconsistent pattern for stop values. In Experiment 1, we

found lower overall stop values for downward compared with upward saccades, *F*(1,11) = 12.27, *p* = .005, *η*^2^ = .53. No significant difference was found between the con- stant and onset conditions, *F*(1,11) = 2.31, *p* = .16, *η*^2^ = .17. We also found a significant

*p*

*p*

interaction effect, *F*(1,11) = 5.85, *p* = .034, *η*^2^ = .35. This interaction effect was charac- terized by lower stop values in the constant compared with the onset condition for downward saccades, and the reverse pattern for upward saccades. Again, none of these significant effects replicated in Experiment 2, *F*s(1,11) < 1, *p*s > .33, *η*^2^s < .09.

*p*

*p*

Taken together, the slope and stop value parameters were not consistently af- fected across experiments by saccade direction or whether the ensuing landing brightness could be prepared for.

# Vertical PLR Asymmetry

Previous work has demonstrated that the PLR is more sensitive to brightnesses pre- sented in the upper compared with the lower visual field (Cai et al., [2023](#_bookmark2); Kardon et al., [1991](#_bookmark7); Strauch et al., [2022](#_bookmark10)). Here, we examined whether a similar effect was apparent in our experiments during the fixation period prior to cue onset. We only report data from the constant conditions because no asymmetries could be present in onset conditions prior to saccade onset (i.e. no brightness patches were yet pre- sented).

Supplementary Figure [1](#_bookmark0) shows a clear vertical asymmetry in the PLR in Experi- ment 1: the brightness patch presented in the upper visual field affects pupil size more strongly. In Experiment 2, we aimed to diminish this effect by presenting bright and dark patches in both the upper and lower visual fields in each trial. This ame- liorated the vertical asymmetry effect (Supplementary Figure [1](#_bookmark0)). This shows that our results were not driven by the vertical PLR asymmetry.

## Upward Downward

200

Land on Bright Land on Dark

100

Pupil size (a.u.)

0

100

200

0 1000 2000 3000

Time (ms)

## Upward

0 1000 2000 3000

Time (ms)

## Downward

200

Land on Bright Land on Dark

100

Pupil size (a.u.)

0

100

200

0 1000 2000 3000

Time (ms)

0 1000 2000 3000

Time (ms)

Supplementary Figure 1: Pupil size over time during the fixation period in the con- stant condition for each experiment. Data from Experiments 1 and 2 are presented in the first and second row respectively. The top row demonstrates the vertical PLR asymmetry, where the brightness presented in the upper meridian affects the PLR more strongly. Note that the effect visually seems to ftip, but that is caused by split- ting on the ultimate landing brightness not by a ftip in vertical asymmetry effect. The bottom row demonstrates that this effect was effectively eliminated in Experi- ment 2.

# Point-by-point PLR difference trace analyses

Our main analyses were based on fitting the exponential decay function to the PLRs obtained in the different conditions. Although this approach is highly informative,

especially because we were interested in when the PLR emerged, we also wanted to examine whether there were differences in the strength of the PLR between di- rections. We here did so without fitting the exponential decay function to the data to be comparable with other work on the PLR.


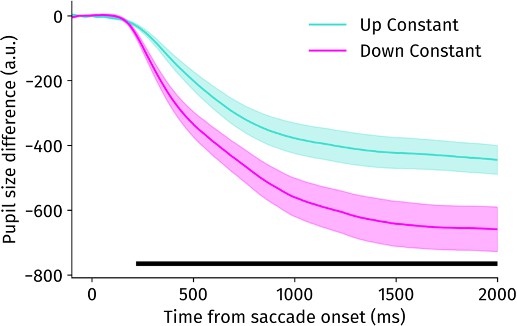

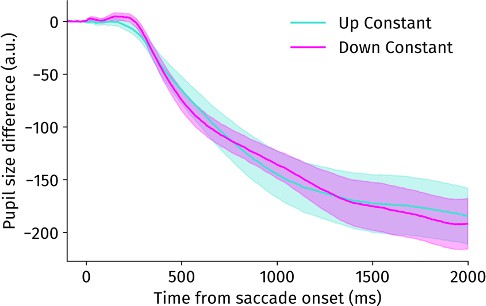


Supplementary Figure 2: PLR difference traces for the constant conditions per ex- periment. The left and right figures represent Experiments 1 and 2, respectively. Horizontal black lines indicate significant clusters, *p*<˙ .001.

To this end, we first compared the evoked PLRs in the constant conditions be- tween up- and downward saccades (Supplementary [Figure 2](#_bookmark1)). We analyzed the PLRs using a cluster-based approach to control for multiple comparisons. In Experiment 1, we observed a significant cluster, indicating the the PLR was stronger for down- compared with upward saccades in the constant conditions (Constant: 220-2000ms, *p* < .001; see black horizontal line in the figure). By contrast, the same analysis yielded no significant clusters in Experiment 2 (*p* = .37). For Experiment 2, we also calculated a Bayes Factor based on the average PLR 0-2000ms after saccadic on- set. This analysis indicated evidence for the null-hypothesis (BF_01_ = 3.47). To check the robustness of these effects, we also calculated the difference between constant and onset conditions separately per condition. This analysis more clearly exam- ines the effect of presaccadic attention on the PLR. These results closely resemble the results obtained above. In Experiment 1, we observed a significant cluster (230- 2000 ms, *p* = .009), while no significant effect was found for Experiment 2 (*p* = .47, BF_01_ = 2.85).

Thus, while Experiment 1 shows a stronger PLR for down- compared with upward

saccades, this effect did not show up in Experiment 2. What could have caused this difference in results? There are a number of methodological changes from Exper- iment 1 to Experiment 2, among these are the more controlled stimuli as well as the position of the eye with respect to the monitor. Another possibility is that this argues for a stronger presaccadic shift for down- compared with upward saccades, and that this is only observable under stronger evoked PLR responses. We consider the latter interpretation unlikely, as the observed PLR responses in Experiment 2 were sensitive enough to pick up on other presaccadic effects such as the latency of the PLR response. Therefore, we argue that the stronger evoked PLR for down- ward saccades could have been caused by the better controlled stimuli or by the eye position with respect to the monitor. More specifically, it is possible that the differences in pupil size between land-dark and land-bright trials between saccade directions (in the constant conditions) could have caused these effects. Differences in ‘baseline’ pupil size are known to modulate pupillary responses strongly (Knapen et al., [2016](#_bookmark8)), and since pupil size differed upon cue onset this could have caused dif- ferences in PLRs between up- and downward saccades ([Figure 1](#_bookmark0)). Indeed, smaller baseline pupil sizes are linked with enhanced subsequent dilations and weaker en- suing constrictions, large baseline pupil sizes show the opposite effects (Knapen et al., [2016](#_bookmark8)). In the data from Experiment 1, for upward saccades land-dark trials had a relatively large pupil size prior to cue onset, and land-bright trials had a rel- atively small pupil size in this interval. This could have caused decreased dilation and constriction responses to the landing brightness in dark and bright trials for upward saccades, respectively. Instead, for downward saccades this baseline effect is reversed, which could have led to increased PLR amplitudes. Therefore, differ- ences in pupil size prior to cue onset caused by a vertical asymmetry of the PLR may account for the PLR amplitude difference in Experiment 1. Indeed, when closely controlling for this in Experiment 2, we did not observe the same difference between up- and downward saccades. Thus, presaccadic attention is shifted both up- and downward, and the strength between these shifts does not seem to differ reliably.

# Saccade landing

As the pupil revealed presaccadic effects only after saccadic onset, we next investi- gated saccade landing. For if saccade landing precision differed between conditions (e.g. directly fixating the landing brightness in one but not the other condition), this might account for some of the observed effects.

To this end, we tested the saccade landing error, saccade under/overshoots and

saccade amplitudes using linear mixed-effects models, Wilkinson Notation: Out- come ∼ Constant/Onset x Direction + (1+Direction|Participant). The results were consistent across experiments. Landing precision was comparable for up- and down- ward saccades in both experiments (Exp. 1: *β* = 0.031, *SE* = 0.383, *t* = 0.082, *p* = .935; Exp. 2: Exp. 1: *β* = 0.788, *SE* = 0.520, *t* = 1.516, *p* = .130). In Experiment 1, we found a slight increase in landing precision when participants saccaded toward down- compared with upward saccades (*β* = 0.267, *SE* = 0.112, *t* = 2.376, *p* = .018), but this effect was not reliable in Experiment 2 (*β* = 0.222, *SE* = 0.125, *t* = 1.781, *p* = .075). The interaction effect was non-significant in both experiments (*t*s < 1.73, *p*s > .08). By contrast, we analyzed the y coordinate landing positions and found that participants tended to under- and overshoot their up- and downward saccades, respectively (Exp. 1: *β* = 2.101, SE = 0.276, *t* = 7.614, *p* < .001; Exp. 2: *β* = 2.419, *SE* = 0.558, *t* = 4.335, *p* < .001 - other effects were non-significant, *t*s < 1.40, *p*s > .16). Saccade amplitudes complemented this findings, downward saccades had larger amplitudes than upward saccades (Exp. 1: *β* = 1.979, *SE* = 0.274, *t* = 7.229, *p* < .001; Exp. 2: *β* = 2.383, *SE* = 0.536, *t* = 4.444, *p* < .001 - other effects

were non-significant, *t*s < 1.50, *p*s > .13).

To summarize, we observed consistent under- and overshoots for up- and down- ward saccades, but the eyes landed as close to the saccade target in both directions, effectively resulting in the same fixated luminance between conditions. Our findings dovetail with the results from Collewijn et al. ([1988](#_bookmark3)) wherein participants system- atically under- and overshot the saccade targets for up- and downward saccades, respectively. However, Hanning et al. ([2022](#_bookmark4), [2024](#_bookmark5)) did not observe differences in saccade amplitudes and landing precision between up- and downward saccades.

Importantly, saccades in the current data still landed on the manipulated bright- ness in the vast majority of trials (Exp. 1: 95.28%, Exp. 2: 78.77%), ensuring that the eyes fixated the landing brightness (also note that we observed robust PLRs in the expected direction in each condition). We also conducted a control analysis for Ex- periment 2, wherein we only included trials where participants landed within the inner circle and the PLR latency effect remained substantial: the PLR emerged ear- lier in constant than in onset trials, *F*(1,11) = 9.65, *p* = .010, *η*^2^ = .47.

*p*

# Saccade offset-locked analyses

To further address the possible inftuence of saccade landing on our results, we also reanalyzed our data when locking to saccade offset instead of saccade onset. This analysis ensures that saccade properties such as duration (i.e. the time the eyes are in-ftight) did not affect our conclusions.

We fit the exponential decay functions as in the main paper, and analyzed the onsets of the PLR after saccade offset. In line with the analyses locked to saccade onset, we observed that the PLR emerged earlier in the constant compared with the onset conditions in both experiments (Exp. 1: *F*(1,11) = 16.45, *p* = .002, *η*^2^ = .60, Exp. 2: *F*(1,11) = 8.88, *p* = .008, *η*^2^ = .49). In both Experiments, the interaction effect between condition and direction were not significant (Exp. 1: *F*(1,11) = 3.90, *p* = .074, *η*^2^ = .26, Exp. 2: *F*(1,11) = 0.70, *p* = .434, *η*^2^ = .06), indicating a comparable difference between the constant and onset conditions between directions. Lastly, we observed an earlier PLR onset for down- compared with upward saccades in Experiment 1, *F*(1,11) = 6.97,

*p*

*p*

*p*

*p*

p = .038, *η*^2^ = .34, but not in Experiment 2, *F*(1,11) = 0.013, *p* = .916, *η*^2^ = .001. This main

*p p*

effect was thus not reliable across Experiments.

Together, these analyses further support the findings when locking to saccade onset. Here, we observed the same effects, leading us to conclude that presaccadic attention is shifted up- and downwards in a comparable manner.

# References

Cai, Y., Strauch, C., Van der Stigchel, S., & Naber, M. (2023). Open-DPSM: An open- source toolkit for modeling pupil size changes to dynamic visual inputs. *Be-* *havior Research Methods*. <https://doi.org/10.3758/s13428-023-02292-1>

Collewijn, H., Erkelens, C. J., & Steinman, R. M. (1988). Binocular co-ordination of human vertical saccadic eye movements. *The Journal of Physiology*, *404*(1), 183–197. <https://doi.org/10.1113/jphysiol.1988.sp017285>

Hanning, N. M., Himmelberg, M. M., & Carrasco, M. (2022). Presaccadic attention en- hances contrast sensitivity, but not at the upper vertical meridian. *iScience*, *25*(2), 103851. <https://doi.org/10.1016/j.isci.2022.103851>

Hanning, N. M., Himmelberg, M. M., & Carrasco, M. (2024). Presaccadic Attention De- pends on Eye Movement Direction and Is Related to V1 Cortical Magnification. *Journal of Neuroscience*, *44*(12). [https://doi.org/10.1523/JNEUROSCI.1023-](https://doi.org/10.1523/JNEUROSCI.1023-23.2023) [23.2023](https://doi.org/10.1523/JNEUROSCI.1023-23.2023)

Hoeks, B., & Levelt, W. J. M. (1993). Pupillary dilation as a measure of attention: A quantitative system analysis. *Behavior Research Methods, Instruments, &* *Computers*, *25*(1), 16–26. <https://doi.org/10.3758/BF03204445>

Kardon, R. H., Kirkali, P. A., & Thompson, H. S. (1991). Automated Pupil Perimetry Pupil Field Mapping in Patients and Normal Subjects. *Ophthalmology*, *98*(4), 485–

496. <https://doi.org/10.1016/S0161-6420(91)32267-X>

Knapen, T., de Gee, J. W., Brascamp, J., Nuiten, S., Hoppenbrouwers, S., & Theeuwes, J. (2016). Cognitive and Ocular Factors Jointly Determine Pupil Responses under Equiluminance. *PLOS ONE*, *11*(5), e0155574. [https://doi.org/10.1371/journal.](https://doi.org/10.1371/journal.pone.0155574) [pone.0155574](https://doi.org/10.1371/journal.pone.0155574)

Mathôt, S., van der Linden, L., Grainger, J., & Vitu, F. (2015). The pupillary light response reftects eye-movement preparation. *Journal of Experimental Psychology: Hu- man Perception and Performance*, *41*(1), 28–35. [https : // doi . org / 10 . 1037/](https://doi.org/10.1037/a0038653) [a0038653](https://doi.org/10.1037/a0038653)

Strauch, C., Romein, C., Naber, M., Van der Stigchel, S., & Ten Brink, A. F. (2022). The orienting response drives pseudoneglect—Evidence from an objective pupil- lometric method. *Cortex*, *151*, 259–271. [https://doi.org/10.1016/j.cortex.2022.](https://doi.org/10.1016/j.cortex.2022.03.006)

[03.006](https://doi.org/10.1016/j.cortex.2022.03.006)
